# Memetic Algorithm-Based Multi-Objective Coverage Optimization for Wireless Sensor Networks

**DOI:** 10.3390/s141120500

**Published:** 2014-10-30

**Authors:** Zhi Chen, Shuai Li, Wenjing Yue

**Affiliations:** 1 College of Computer, Nanjing University of Posts and Telecommunications, No.9, Wenyuan Road, Yadong new District, Nanjing 210023, China; E-Mail: acm@njupt.edu.cn; 2 Jiangsu High Technology Research Key Laboratory for Wireless Sensor Networks, No.66, New Mofan Road, Gulou District, Nanjing 210003, China; 3 State Key Laboratory for Novel Software Technology, Nanjing University, No.163, Xianlin Road, Qixia District, Nanjing 210023, China; 4 Key Lab of Broadband Wireless Communication and Sensor Network Technology, Ministry of Education, No.66, New Mofan Road, Gulou District, Nanjing 210003, China; E-Mail: yuewj@njupt.edu.cn

**Keywords:** sensor networks, coverage algorithm, memetic algorithm, multi-objective optimization

## Abstract

Maintaining effective coverage and extending the network lifetime as much as possible has become one of the most critical issues in the coverage of WSNs. In this paper, we propose a multi-objective coverage optimization algorithm for WSNs, namely MOCADMA, which models the coverage control of WSNs as the multi-objective optimization problem. MOCADMA uses a memetic algorithm with a dynamic local search strategy to optimize the coverage of WSNs and achieve the objectives such as high network coverage, effective node utilization and more residual energy. In MOCADMA, the alternative solutions are represented as the chromosomes in matrix form, and the optimal solutions are selected through numerous iterations of the evolution process, including selection, crossover, mutation, local enhancement, and fitness evaluation. The experiment and evaluation results show MOCADMA can have good capabilities in maintaining the sensing coverage, achieve higher network coverage while improving the energy efficiency and effectively prolonging the network lifetime, and have a significant improvement over some existing algorithms.

## 1. Introduction

Wireless Sensor Networks (WSNs) are self-organized networks consisting of sensor nodes with the ability of sensing, processing and wireless communicating [1]. Coverage control is one of the most fundamental research issues in sensor networks, and studies how well a sensor network will monitor a field of interest with the proper node deployment [2,3]. Sensor nodes often have constrained resources and it is sometimes difficult to recharge their energy, and thus coverage sustainability in such sensor networks cannot be guaranteed. How to balance the network energy consumption in coverage control is an important issue, which can be modeled as a multi-objective optimization problem of prolonging the network lifetime and improving network coverage according to the characteristics of WSNs [4].

Multi-objective optimization problems involve two or more conflicting objectives and have not one optimal solution but many solutions which form the Pareto front representing a tradeoff of one objective against the other. In most applications, the goal of solving the multi-objective optimization problems is to compute an approximation of the Pareto front. Computational Intelligence (CI) and evolutionary algorithms provide adaptive, flexible and robust mechanisms that exhibit intelligent behavior to solve the multi-objective optimization problems of coverage control in complex and dynamic environments like WSNs [5]. Habib modeled the coverage problem with two sub-problems: floorplan and placement, and used the genetic algorithm (GA) to search the optimal coverage in WSNs [6]. Ozturk *et al.* applied artificial bee colony algorithm to the dynamic deployment of the sensor networks and obtained better deployments for WSNs than the particle swarm optimization algorithm [7,8]. The aforementioned articles emphasized to improve the coverage rate and did not specifically take into account network lifetime and energy balance in WSNs. Memetic algorithms are computational intelligence structures and a class of stochastic global search heuristics where evolutionary algorithms are combined with multiple and various local search heuristics in order to address such optimization problems as those in WSNs [9–11].

This paper presents a multi-objective coverage optimization algorithm based on memetic algorithm for WSNs, namely MOCOAMA, which considers the coverage degree, node utilization, node residual energy, and solves the 1-coverage multi-objective problem for WSNs [12–15], and finally gets the optimal deployment of network coverage.

The rest of the paper is organized into five sections: Section 2 briefly introduces related work. Section 3 discusses the multi-objective optimization coverage problem of WSNs. Section 4 presents the key schemes for the proposed coverage algorithm for WSNs. Section 5 describes the multi-objective optimization coverage algorithm based on memetic algorithms. The simulation experiments and evaluation are given in Section 6. Finally, the conclusions are offered in Section 7.

## 2. Related Work

Konstantinidis *et al.* proposed a memetic algorithm (MA)-based solution of energy-aware topology control (ToCMA) for WSNs using a combination of problem-specific light-weighted local searches and genetic algorithms [11], which can provide a better performance than the classical minimum spanning tree solutions. In ToCMA, the composing entities of a chromosome or genes are the power of sensor nodes; the fitness function of a solution is the sum of the power assigned to each gene, and repair and improvement methods are employed to refine solutions.

Ferentinos *et al.* used a memetic algorithm to dynamically optimize the design of WSNs and considered different constraints such as application-specific requirements, communication constraints and energy consumption [14]. Their work showed that the hybridization of the original GA with the local search operations presented in memetic algorithms brought some improvement on the performance of the design process.

Jiang *et al.* used memetic algorithms to implement energy-efficient coverage control in cluster-based WSNs (CoCMA) [15], which contains a memetic algorithm-based schedule for sensor nodes and a wake-up scheme. In CoCMA, the coverage solutions are represented by binary strings, and the status of a node is represented by an allele of a chromosome. CoCMA can prolong the network lifetime while maintaining coverage preservation of WSNs with no sensing error, and has a significantly improved performance compared with the LEACH [16], LEACH-Coverage-U [17], *etc*.

Ting *et al.* proposed a memetic algorithm to solve the set k-cover problem of WSNs, which has the effectiveness and efficiency of extending the network lifetime [18]. In MA, a chromosome represents the sequence in which all sensor nodes are collected to form covers, and the fitness value of a chromosome is the sum of coverage contributions of all sensor nodes and can enhance the differentiation of promising chromosomes.

Arivudainambi *et al.* proposed a knowledge added improved memetic algorithm (iMA) for target coverage in WSNs, which is concerned with exploiting all available knowledge and demonstrated the effectiveness in extending the lifetime of WSNs [19]. iMA encodes the candidate solution as the chromosome represented by a matrix in which row represents the sensor nodes, and column represents the targets.

In this paper, our proposed MOCOAMA uses the coverage optimization framework of CoCMA [15], MA [18] and, iMA [19], but it deals with the coverage problem of WSNs with the sensing error, divides the target area with the virtual cells as the basis of chromosome representation, and randomly and alternately selects some local search algorithms to achieve the dynamic local search.

## 3. Multi-Objective Coverage Optimization Problem

In this paper, we assume that sensor nodes are randomly and uniformly distributed over the target area, with the same physical structure, communication capacity, sensing range, initial limited energy and computing capacity. The sink node or the base station (sink) is assumed to have unlimited energy with plenty of power supplies. The sink or every sensor node has a unique identifier, and can get its own location information and communicate with its neighbor nodes usually using normal power or with each other through power control.

A target field *D* is a two-dimensional plane divided into *M* × *N* virtual cells. The points of interest are distributed in *D* and there exists at most one point of interest in one virtual cell. A set of sensor nodes *T* are deployed over *D* where *T* ={*t_1_*, *t_2_*,…, *t_n_*}, *t_i_* = (*x_i_*, y*_i_*, r*_i_*), *n* is the number of sensor nodes, *i* ∈ [1, *n*], (*x_i_*, y*_i_*) is the coordinate of the sensor node *t_i_*, and r*_i_* is the maximum ideal sensing radius of *t_i_* The coverage field of *t_i_* is a circle area with (*x_i_*, y*_i_*) as the center and r*_i_* as the radius. The Euclidean distance between *t_i_* and the point of interest *c* whose coordinate is (*x*, y) in *D* is:

(1) d(ti,c)=(xi−x)2+(y−iy)2

If the set of virtual cells sensed by the sensor node *t_i_* is *s_i_*, the coverage area of all sensor nodes is:

(2) $$S(T)=|\overset{n}{\underset{i=1}{\cup }}\gamma_{i}s_{i}|$$

where *γ_i_* is the decision variable to be determined by the coverage algorithm, *γ_i_* = 1 if *t_i_* is in the state of activation, and *γ_i_* = 0 if *t_i_* is in the state of inactivation.

The probability of sensing the point of interest *c* by the sensor node *t_i_* in *D* is:

(3) $$p(t_{i},c)=\left\{ \begin{array}{ll} 0 & r_{i}\leq d(t_{i},c)\\ e^{-\lambda \times \frac{d(t_{i},d)-r_{i}-r_{e}}{r_{i}-d(t_{i},d)}} & r_{i}-r_{e}<d(t_{i},c)<r_{i}\\ 1 & r_{i}-r_{e}\geq d(t_{i},c) \end{array}\right.$$

where *r_e_* is the sensing error value of the sensor nodes, r*_i_* has the same value for all sensor networks assumed in this paper and *λ* is the sensing attenuation coefficient.

As long as the point of interest *c* is effectively sensed by a sensor node, it can be said that *c* is covered by the network. If a set of points of interest (*i.e.*, *c*_1_, *c*_2_,…,*c_k_*, *k* ≤ *M* × *N*) at the same time is sensed by *t_i_*, we get:

(4) |si|=k(1−∏j=1k(1−p(t,icj)))

We define sensing coverage degree or coverage degree as the percentage of the field area covered by sensor nodes in the monitored field, which reflects the actual network sensing ability of monitoring a given field of interest or the points of interest. According to Equation (2), the coverage degree of sensor networks deployed in *D* is:

(5) $$\varpi (T)=\frac{S(T)}{M\times N}=\frac{\left|\overset{n}{\underset{i=1}{\cup }}\gamma_{i}s_{i}\right|}{M\times N}$$

According to Equation (4), we can get:

(6) ϖ(T)≤∑i=1nγi|si|M×N≤∑i=1nγik(1−∏j=1k(1−p(t,icj)))M×N

If *k* = *M* × *N*, we can get:

(7) ϖ(T)≤∑i=1nγi(1−∏j=1M×N(1−p(t,icj)))

We define the objective optimization model of the network coverage as:

(8) $$\{\begin{array}{l} \min\overset{n}{\underset{i=1}{\text{∑}}}\gamma_{i}\\ \max\varpi (T) \end{array}$$

In WSNs, due to the limited energy of sensor nodes, reducing the energy consumption of some nodes and balancing the energy consumption of the entire network can effectively save the limited energy of the network and extend the network lifetime. Under the premise of guaranteeing some certain coverage degree, we may reduce the number of sensor nodes in working and deactivate the redundant nodes to reduce the energy consumption as much as possible to increase the network lifetime.

We define node utilization as the ratio of the number of cells having active sensor nodes to the total cell number in the target area given by:

(9) $$U(T)=\frac{\left||T|\right|}{M\times N}$$

where ║T║ is the number of cells having active sensor nodes and sensor nodes are deployed in the target field *D* which is divided into *M* × *N* virtual cells.

To extend the lifetime of sensor networks, we also consider the residual energy of WSNs in the target field *D*. If *E*_i_ is the residual energy of the sensor node *t_i_*, the energy distribution of the network is expressed as:

(10) $$E(T)=\frac{\frac{1}{n}\overset{n}{\underset{i=1}{\text{∑}}}{\left({\text{E}}_{i}-\frac{1}{n}\overset{n}{\underset{i=1}{\text{∑}}}{\text{E}}_{i}\right)}^{2}}{{\left(\frac{1}{n}\overset{n}{\underset{i=1}{\text{∑}}}{\text{E}}_{i}\right)}^{2}}=\frac{n\overset{n}{\underset{i=1}{\text{∑}}}{\left({\text{E}}_{i}-\frac{1}{n}\overset{n}{\underset{i=1}{\text{∑}}}{\text{E}}_{i}\right)}^{2}}{{\left(\overset{n}{\underset{i=1}{\text{∑}}}{\text{E}}_{i}\right)}^{2}}$$

where *E*(*T*) reflects the degree of energy difference in the network; if its value is smaller, the energy difference between sensor nodes may be smaller, and the residual energy of the sensor nodes may be higher.

To increase the network lifetime, we may not only use as few nodes as possible to reduce energy consumption, but also make sure to select the nodes with balanced energy consumption and a higher residual energy. Therefore, we define the objective optimization model of the energy consumption as:

(11) $$\{\begin{array}{l} \min\alpha \times U(T)+\beta \times E(T)\\ \max\varpi (T) \end{array}$$

where *α* is the node utilization weighting coefficient, *β* is the energy balance weighting coefficient and *α* + *β* = 1.

According to Equations (8) and (11), in MOCOAMA, we pay attention to Pareto optimal solutions of the multi-objective coverage optimization problem of WSNs formulated as:

(12) $$\begin{array}{l} \{\begin{array}{l} \min\overset{n}{\underset{i=1}{\text{∑}}}\gamma_{i}\\ \begin{array}{l} \max\varpi (T)\\ \min\alpha \times U(T)+\beta \times E(T) \end{array} \end{array}\\ s.t.\gamma_{i}=0\;or\;1,\text{i}\in [1,\text{n}] \end{array}$$

## 4. Multi-Objective Coverage Optimization Designs

A memetic algorithm uses the notion of meme(s) as units of information encoded in computational representations [20], which is a combination of global search and local search, and the objects of genetic manipulation are not any individuals in the population space, but some locally-optimal representatives elected by local search and from the local area. Using the inherent parallelism of memetic algorithm, we can greatly accelerate the rate of convergence of the multi-objective coverage optimization algorithm for WSNs.

### 4.1. Encoding Rule

Encoding is a mapping from the problem space to the solution space, but memetic algorithm cannot directly process the solution data of the solution space, so before searching, the variables of the solution space must be mapped into the data structures of evolutionary space, namely chromosomes. In MOCOAMA, when the sink divides the target area into *M* × *N* cells, each chromosome represents a deployment solution of WSNs with the form of an *M* × *N* matrix given in Equation (13):

(13) $$ℵ=\left[\begin{array}{cccc} {\text{X}}_{1,1} & {\text{X}}_{1,2} & \cdots & {\text{X}}_{1,N}\\ {\text{X}}_{2,1} & {\text{X}}_{2,2} & \cdots & {\text{X}}_{2,N}\\ \vdots & \vdots & \ddots & \vdots \\ {\text{X}}_{M,1} & {\text{X}}_{M,2} & \cdots & {\text{X}}_{M,N} \end{array}\right]$$

In each chromosome **ℵ**, allele *X_i,j_* denotes one of the *n* sensor nodes covering the virtual cell of (i,j) where *X_i,j_* ∈[0,n]; if *X_i,j_* = 0, the virtual cell of (i,j) is not covered by any sensor node. The sensor nodes in one cell have one of three states: “sleeping” state, “detecting” state and “working” state; the “working” state is the state of activation, and other two states are the states of inactivation. Sensor nodes covering one cell update their remaining energy in real time, and the sink chooses one sensor node as the working node which has shorter distance from the center of the virtual cell or has more residual energy and has a priority right to being the working node. After collecting the information on locations and residual energy of all nodes covering each cell, the sink computes the probability of being a working node for each sensor node. The node with greater probability will be elected as the working node covering one cell, or a node will be randomly selected as the working node from those nodes with the same probability, and other nodes will turn into being the sleeping state from the detecting state. In order to balance the energy consumption of each node, re-election of the working node covering one cell will be requested under the control of the sink when the remaining energy of the working node is less than the average energy of sensor nodes covering one cell.

In MOCOAMA, only one of the sensor nodes is elected in its virtual cell; one virtual cell is covered at least one sensor node at the different time unless it cannot be covered by any sensor node. Therefore, from each chromosome, given the WSNs of *T* ={*t*_1_, *t*_2_,…, *t_n_*}, we can get some sets of sensor nodes in working state and one set of sensor nodes can be described as:

(14) $$\Psi =\overset{i=M,j=N}{\underset{i=1,j=1}{\cup }}\left\{X_{i,j}\right\}=\overset{n}{\underset{i=1}{\cup }}\gamma_{i}t_{i}$$

where *γ_i_* = 1 if the sensor node *i* is in working state and *γ_i_* = 0 if otherwise.

According to Equations (13) and (14), we can get one of the decision solutions of deploying WSNs given by:

(15) $$\Gamma =\overset{n}{\underset{i=1}{\cup }}\gamma_{i}$$

### 4.2. Fitness Function

In MOCOAMA, the fitness of each chromosome may be evaluated according to the values of the multiple optimization sub-goals. We apply Pareto ranking [21] as the fitness value evaluation scheme in searching for a set of Pareto-optimal solutions of multi-objective coverage optimization for WSNs. The allocation order of each non-dominated individual in current population is 1; the allocation order of any other individual is the number of dominant individuals plus 1; the formula is as follows:

(16) $$R({ℵ}_{i})=|\{{ℵ}_{j}|{ℵ}_{j}\in P,{ℵ}_{j}≻{ℵ}_{i},\forall {ℵ}_{i}\in P\}|$$

where **ℵ***_i_* is any individual of the population *P*, ≻ means the dominance relationship between two individuals, *R*(**ℵ***_i_*) means the number of individuals which dominate **ℵ***_i_* in population P. The allocation order of **ℵ***_i_* is *R*(**ℵ***_i_*) +1. If the individual **ℵ***_i_* is closer to the optimal solution, the order number of **ℵ***_i_* is smaller. When **ℵ***_i_* is a non-dominated solution, the order number of **ℵ***_i_* is 1. According to Equations (12) and (16), the fitness function in MOCOAMA is:

(17) $$f({ℵ}_{i})={\left(\delta_{1}(\alpha \times U(T)+\beta \times E(T))-\delta_{2}\varpi (T)\right)}^{-(R({ℵ}_{i})+1)\times \sum_{i=1}^{n}\gamma_{i}}$$

### 4.3. Local Search Strategy

The local search strategy of memetic algorithms is a process of screening the excellent individuals in the local area. The combination of global search algorithms and local search algorithms often shows good convergence and strong global search capability in solving the multi-objective optimization problem, but there are no uniform standards and guides in the choice of local search strategies, as well as the position, mild and frequency, so we need to discover the local search strategy which is suitable for the unresolved problem. Synthesizing the advantages of a variety of local search algorithms can make the process of the local search converge faster and have higher solution quality by designing specific local search access policies.

In order to achieve dynamic local search, we propose some self-adaption scheduling rules of the local algorithm. In every iteration of MOCOAMA, some algorithms from the pool of local search algorithms are randomly and alternately selected to make local search and get diverse solutions. Then the sink searches *m* searched nearest points from the initial point of the local search and the situation of solution fitness improvement corresponding to the local search algorithms used in the *m* points is evaluated. The one of the local search algorithms having the most obvious improvement on fitness is eventually used in the multi-objective optimization. The proposed rules learn from the search history information to regulate the local search strategy with adaptive, self-learning features.

The pool of local search algorithms is a collection of many local search algorithms, including the tabu search algorithm [22], hill climbing search algorithm [23] and adaptive directional local search strategy [24]. Tabu search is an optimization algorithm for simulating human intelligence, which mimics the human memory function, saves the searched optimal solution to the tabu table in the process of solving, and marks the solutions to avoid repeating the same search in order to gain a broad search range [22]. The hill-climbing algorithm is a heuristic search algorithm based on greedy search strategy, which selects a random solution as the current solution in the solution space and compares with the solutions in the neighborhood scope one by one until you find a local optimal solution [23]. The adaptive directional local search strategy dynamically adjusts the neighborhood radius and/or local search probability, depending on the relative local and global effectiveness of evolutionary operators and the local search operator [24].

## 5. Memetic Algorithm Based Multi-Objective Coverage Optimization

In this paper, MOCADMA uses the dynamic local search strategy and a local algorithm scheduling mechanism to adapt to the selection of local search algorithms; and increases the optimal storage strategy to accelerate the convergence speed of the algorithm. The pseudo code of MOCADMA is described as follows:
*Pseudo code of MOCADMA**Given information : the target field D*, *the points of interest* {*c*_1_,*c*_2_,…,*c_k_*},*        the sink node (Sink), the set of sensor nodes T ={t_1_,t_2_,…,t_n_* },*        the candidate population* (*CP*), *the optimal population* (*P*)*        the candidate population size* (*pts*), *the optimal population size* (*pos*),*        the crossover probability* (*pcross*), *the mutation probability* (*pmutation*)*        maximum iterations* (*mi*), *parameters used in the fitness function**step*(A) : *Deploying T over D** for i* ← *1 to n do**  Sink* ← (*location_i_, r_i_, re_i_*, *residual _ energy_i_*)* end for**step*(*B*):*D → M × N virtual cells**step*(*C*):* for i ←* 1 *to n do**  for j* ← 1 *to k do**   Calculate p*(*t_i_*,*c_j_*)*  endfor** endfor** for i ←* 1 *to n do**  calculate* | *s_i_* |* endfor** repeat**  for i ←* 1*to M do**   for j ←* 1 *to N do**    X_i_*_,_
*_j_ ← t= RouletteWheelSelect* (*T, i, j*)*   endfor**  endfor**  *ℵ, ← *EncodeChromosome*(
$\text{Matri}x_{i=1,j=1}^{i=M,j=N}$(*X_i_*_,_
*_j_*))*  if* (ℵ ∉ *CP*) *then**   CP ←* ℵ*  endif** until the number of individuals in CP reaches pts**  for i* ←1 *to pts do**   Calculate f* (ℵ*_i_*)*  endfor**  repeat**   Select the Pareto* – *optimal individual in CP and add it into P until the number of individuals in P reaches pos**repeat**step*(*D*) : *for i* ←1 *to NumofCrossover do**   ChromoA* ← *Select*(*P*)*   ChromoB* ← *Select*(*P*)*   OP* ← *OP* +*Crossover*(*A*, *B*, *pcross*)*  endfor**step*(*E*) : *for i* ←1 *to NumofMutation do**   ChromoA* ← *Select*(*OP*)*   OP* ← *OP* + *Mutate*(*A*, *pmutation*)*  endfor**step*(*F*) : //*randomly and alternately* s*elect some local search algorithms to make local search**   P_1_* ← *SelectTabuSearch(P* ⋃ *OP) //* example 1*   P_2_* ← *HillClimbingSearch(P*⋃*OP) //* example2*   P3* ← *AdaptiveDirectionalSearch*(*P* ⋃ *OP) //* example 3*   CP*← *Evaluate*(*P*_1_,*P*_2_,*P*_3_) // *select the optimal candidate solution**step*(*G*) :*repeat**    Select the Pareto* - *optimal individual in CP and add it into P until the number of individuals in P reaches pos**   until the number of iterations reaches mi**step*(*H*) : *Get current optimal deployment solution from P**step*(*I*) : *Execute current optimal deployment solution over T*

MOCADMA includes iterated evolutionary operations of memetic algorithm and dynamic local search strategy, which is described in detail as follows:

(A) The Sink Collects the Information of Sensor Nodes
  After initial uniform deployment, every sensor node is in the “detecting” state and sends its location information (*#location*), maximum ideal sensing radius (*#r*), sensing error value (*#re*) and energy information (*#residual_Energy*) to the sink by flooding communication.
(B) The Sink Divides the Network
  The sink divides the monitored target field into *M* × *N* virtual cells (or units) according to the locations of the sensor nodes; each sensor node belongs to only one cell but can cover numerous cells; one cell may have many sensor nodes.
(C) The Sink Generates the Initial Population, Calculates the Fitness Values and Saves the Optimal Solutions
  The sink calculates the probability of sensing points of interest by sensor nodes according to Equation (3) and the numbers of virtual cells sensed by sensor nodes according to Equation (4) using the information (*#location*, *#r*, *#re*) of every sensor node, repeatedly selects one node in every cell as the working node by roulette wheel selection that takes the number of the sensed virtual cells as the weight of every node, and generates the deployment solutions which form the initial candidate population *CP* of chromosomes. Then the sink gets the decision variables of sensor nodes from the chromosomes and calculates the fitness value of individual chromosome **ℵ***_i_* in the initial population according to Equation (17) using the information (*#residual_energy*) of sensor nodes, selects the chromosomes with higher fitness value as the local Pareto-optimal solutions, and then saves these solutions in the current optimal population *P*. For the local optimal solution, if the population *P* does not contain **ℵ***_i_* and **ℵ***_i_* is not dominated by the existed solutions in *P*, the sink adds **ℵ***_i_* to *P*. At the same time, the Euclidean distance of the local best individual **ℵ***_i_* to other individuals in the external population space is calculated, and the closer individual is substituted for **ℵ***_i_*.
(D) Crossover
  The sink takes multi-point crossover through exchanging the statuses of nodes in different local optimal chromosomes represented as the matrices. Two individual chromosomes in the current optimal population *P* are randomly and continuously selected and exchange one random row with the given crossover probability; if this recombination generates two new individuals, they will be added into the offspring population *OP*.
(E) Mutation
  To maintain the diversity of solutions, the sink generates a variety of solutions through randomly changing the status of the nodes in the virtual cells. The mutation operator is applied to all individual chromosomes in *OP*, and every allele in one chromosome is modified with the given mutation probability; if this recombination generates a new individual, it will be added into *OP*.
(F) Dynamic Local Search
  Some algorithm from the pool of local search algorithms, including the tabu search algorithm [22], hill climbing search algorithm [23] and adaptive directional local search strategy [24] is randomly and alternately selected to make local search in *P*⋃*OP*, and every local search algorithm generates its local population; then the sink compares the average fitness values of the same number of sample individuals around the initial point of the local search in those local populations, gets the local search algorithm that has the highest average fitness value, and selects its local population as the current candidate population *CP*.
(G) The Sink Calculates the Fitness Values and Saves the Optimal Solutions
  The sink calculates the fitness value of individual chromosome **ℵ**_i_ in the current candidate population *CP*, selects the local Pareto-optimal solutions, and then saves these solutions in *P*.
(H) The Sink Determines the Current Optimal Deployment Solution
  When the number of iterations has reached the predetermined maximum threshold, the sink finishes determining the current optimal deployment solution having the highest fitness value; otherwise, MOCADMA continues to run from *(D)*.
(I) The Sink Broadcasts the Deployment Solution
  The sink broadcasts the optimal network deployment solution to each sensor node, and informs each node in its cell responsible as the working node or the non-working node. The working nodes are in the “working” state covering the target area with the optimal deployment solution, and the non-working nodes are alternate in the “sleeping” or “detecting” state in a period of time. The sink monitors the network and evaluates the energy consuming of every sensor node. When the next round threshold time that may be decided by the routing protocol has arrived, or the remaining energy of one working node is less than the average energy in one cell, another new round comes and MOCADMA continues to run from *(D)*.

## 6. Experiments and Evaluation

We use Matlab to perform the simulation experiments of MOCADMA in which the sink is placed at the center of the target area. In fact, the sink may be deployed at any place in the area, but the energy consumption will be distributed over the field evenly if the position of the sink approximates the geometric center of the target area [15]. An energy consumption model [15,16,25] is used in the simulation experiments. In the energy consumption mode, the transmission energy *E_TX_* for transmitting *K* bits of information between two sensor nodes is calculated by Equation (18); the consumed energy *E_RX_* for receiving *K* bits of information by one sensor node is calculated by Equation (19); when the clustered routing algorithm is applied to the WSNs, the energy consumption of one cluster head [16] is calculated by Equation (20):

(18) $$E_{TX}=K\times E_{elec}+\varepsilon_{fs}\times K\times d^{2}$$

(19) $$E_{RX}=K\times E_{elec}$$

(20) $$E_{CH}=(\frac{n}{C}-1)\times K\times E_{\text{elec}}+K\times E_{\text{elec}}+\frac{n}{C}\times K\times E_{DA}+\varepsilon_{\text{amp}}\times K\times d_{\text{toSink}}^{4}$$

where *n* is the number of sensor nodes, *c* is the number of clusters, *d_toSink_* is the distance between the cluster head and the sink, *E_elec_* is the energy consumed by the electronics in the transmitter or receiver, *ε_fs_* is the energy consumption of the signal power amplifier per square meter, *E_DA_* is the energy consumption of processing one-unit bit data, and *ε_amp_* is the energy consumption of transmitting one-unit bit data to the sink node.

To evaluate the performances of MOCADMA, simulation results are compared to those of CoCMA [15], MA [18] and iMA [19] using the same parameters shown in Table 1, and all the results are from the experiments repeated 30 times.

**Figure 1.**
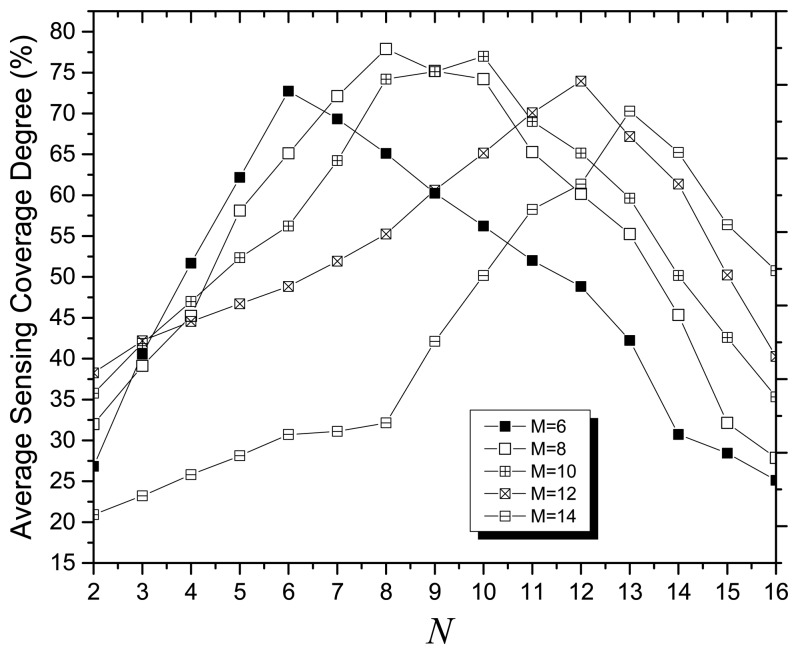
Average SCD *versus* different dividing of virtual cells.

**Figure 2.**
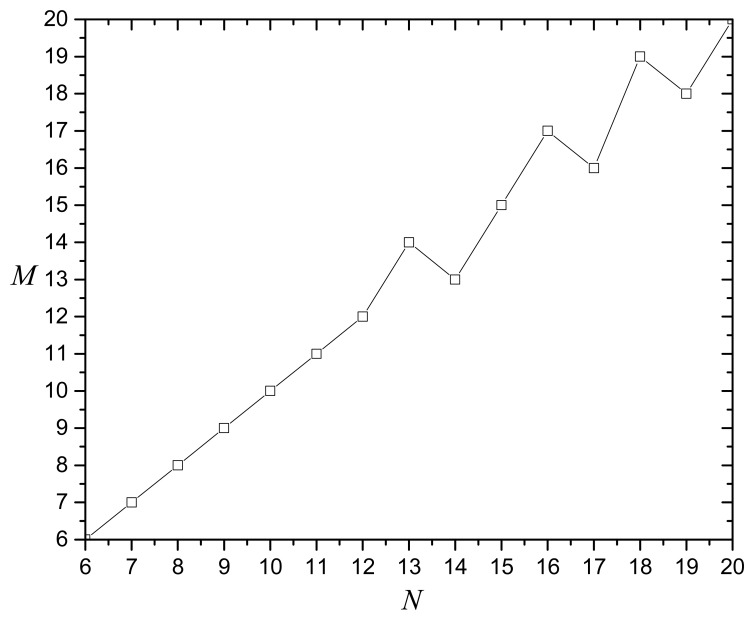
The best dividing of virtual cells having maximum average SCD.

For MOCADMA, the *M* × *N* virtual cells dividing the target area at the initial stage bridge the problem space of WSNs and the solution space of chromosomes. In the simulation experiments, we consider 400 sensor nodes with no sensing error (r*_e_* = 0) and 64 points of interest randomly and uniformly deployed in a 100 m × 100 m target area. Figure 1 shows the average sensing coverage degree (SCD) *versus* different dividing of virtual cells and indicates that the average SCD has the maximum value at *N* = 6 when *M* = 6, *N* = 8 when *M* = 8, *N* = 12 when *M* = 12, and *N* = 13 when *M* = 14. As Figure 2 shows, we can get the best dividing of virtual cells having maximum average SCD if *M* ≈ N.

**Figure 3.**
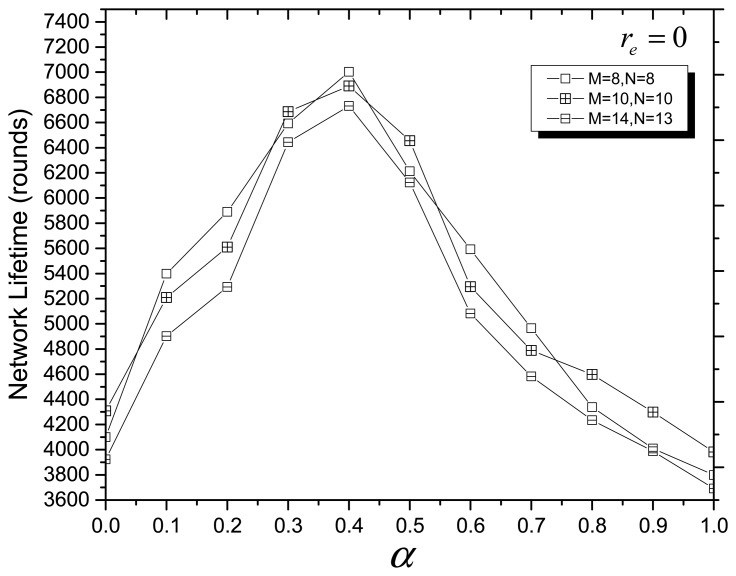
Network lifetime *versus* different values of *α* with r*_e_* = 0.

**Figure 4.**
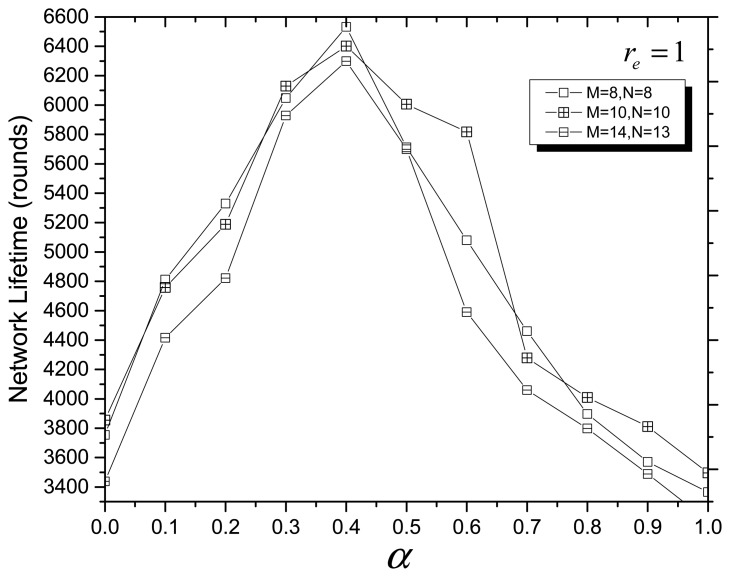
Network lifetime *versus* different values of *α* with r*_e_* = 1.

In the fitness function of MOCADMA, *α* and *β* (*β* = 1 − *α*) are the node utilization weighting coefficient and the energy balance weighting coefficient, respectively. In the next simulation experiments, we assume that *N* = 8, *M* = 8, or *N* = 10, *M* = 10, or *N* = 13, *M* = 14. Figures 3 and 4 depict the network lifetime (rounds) *versus* different values of *α* when r*_e_* = 0 and r*_e_* = 1, respectively, and we can see that the network has a maximum lifetime (rounds) when *α* ≈ 0.4 (*β* ≈ 0.6).

**Figure 5.**
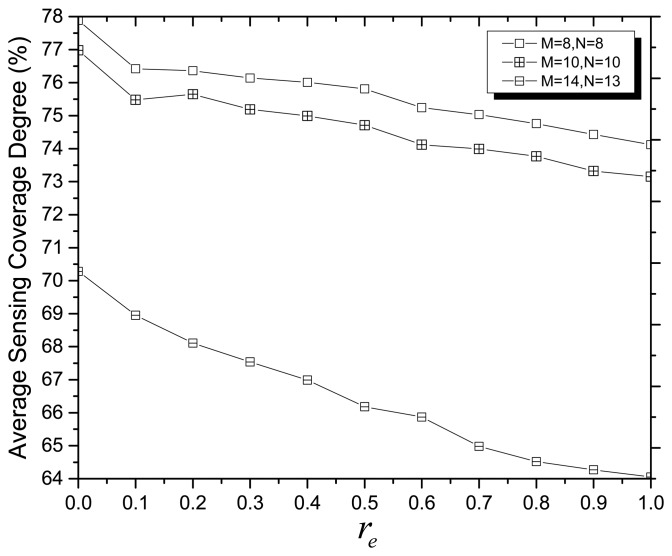
Average SCD *versus* different values of r*_e_*.

In a hostile environment, the sensing error often exists in the applications of WSNs, so we consider it (r*_e_*) in computing the probability of sensing the point of interest by one sensor node eventually used in the fitness function of MOCADMA. Figure 5 shows the average SCD *versus* different values of r*_e_* and indicates that the average SCD decreases with the increase of r*_e_*. It is clearly seen that the more uncertainty from the sensing error causes more difficulties for network coverage.

**Figure 6.**
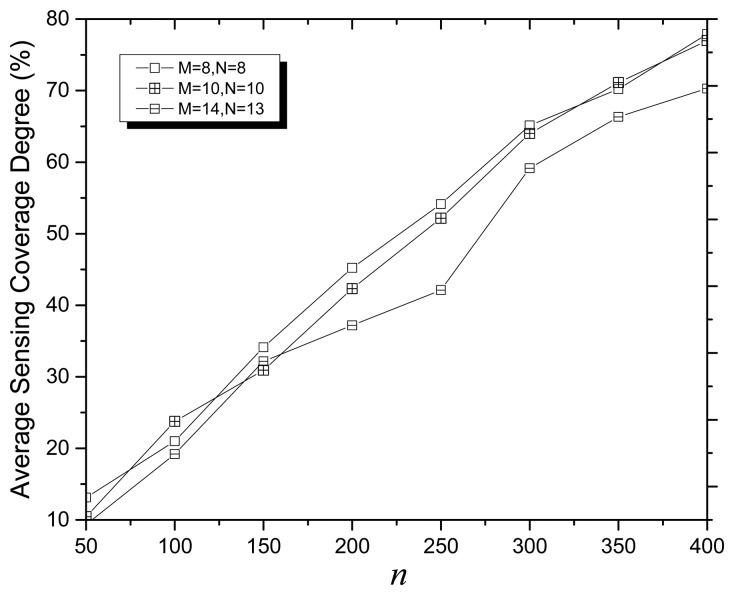
Average SCD *versus* network size with the fixed number of points of interest.

Figure 6 presents the average SCD *versus* network size with the fixed number of points of interest where *α* = 0.4, r*_e_* = 0. As Figure 6 shows, when 64 points of interest randomly and uniformly are deployed in the 100 m × 100 m target area, and the number of deployed sensor nodes varies from 50 to 400, the average SCD increases with the increase of network size (*n*).

**Figure 7.**
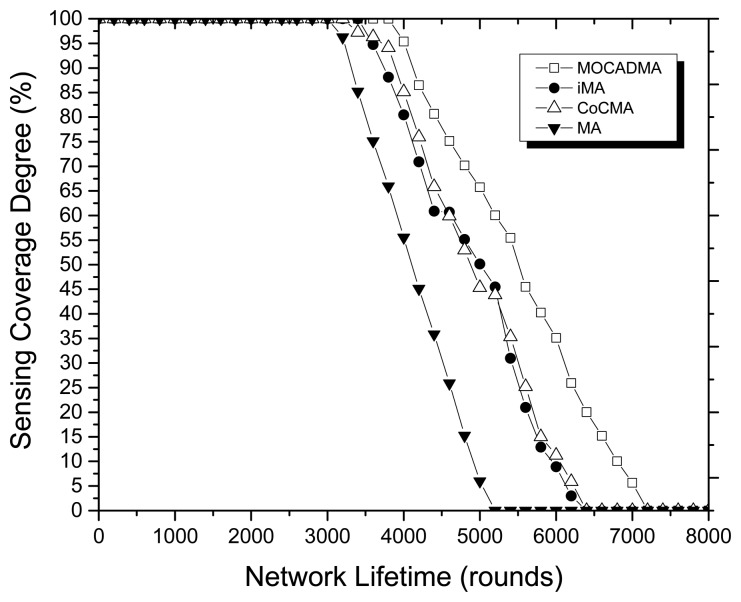
SCD *versus* network lifetime in rounds.

In the next simulations, we verify the feasibility of MOCADMA in WSNs and consider 400 sensor nodes and 64 points of interest randomly and uniformly deployed in the 100 m × 100 m target area. To evaluate the performance of the MOCADMA, an LEACH-based clustered routing protocol [26] is applied to the WSNs, and CoCMA [15], MA [18] and iMA [19] are re-implemented. In addition, the design of CoCMA, MA and iMA didn't allow the sensing error, so we only consider the WSNs with no sensing error (r*_e_* = 0) in the simulation experiments. Figure 7 depicts the SCD *versus* network lifetime in rounds (*α* = 0.4 in MOCADMA). We note that all the algorithms have good capabilities in maintaining the sensing coverage, but MOCADMA has maintained the sensing coverage degree at 100% until the 3800th additional round which is a significant improvement over other algorithms. As Figure 7 shows, the network lifetime is prolonged to 7000 more rounds by the MOCADMA, which lasts about 800 rounds longer compared to CoCMA and iMA.

**Figure 8.**
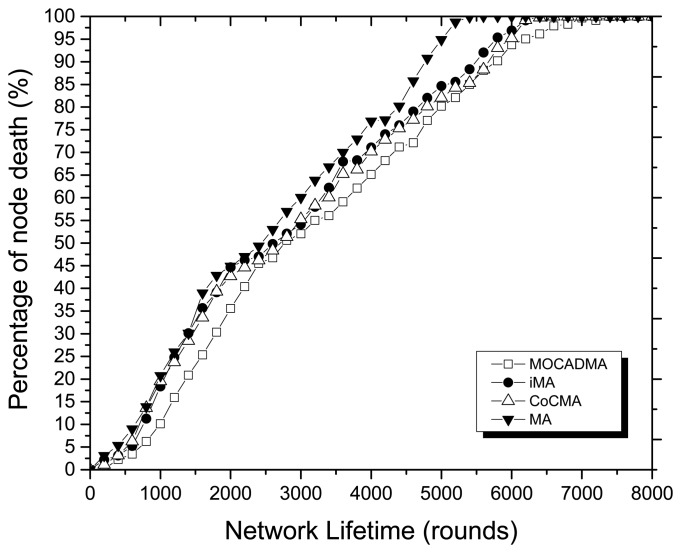
Percentage of node death *versus* network lifetime in rounds.

Figure 8 displays the percentage of node death *versus* network lifetime in rounds (*α* = 0.4 in MOCADMA). The MOCADMA, CoCMA, iMA, and MA lose their 50% of nodes at the 5516th, 5142nd, 4889th, and 4103rd round, respectively. The simulation result demonstrates that the proposed MOCADMA significantly prolongs the lifetime of the network compared to other three methods. The proposed MOCADMA divides the WSNs at the early stage and helps form the clusters earlier than other three methods that save much energy, get the multi-objective Pareto-optimal solutions of network coverage by the fitness function presented in Equation (17) that has a quick-convergence characteristic, so the longer network lifetime can be obtained. The simulation results show that MOCADMA can achieve higher network coverage while improving the energy efficiency and effectively prolonging the network lifetime.

**Figure 9.**
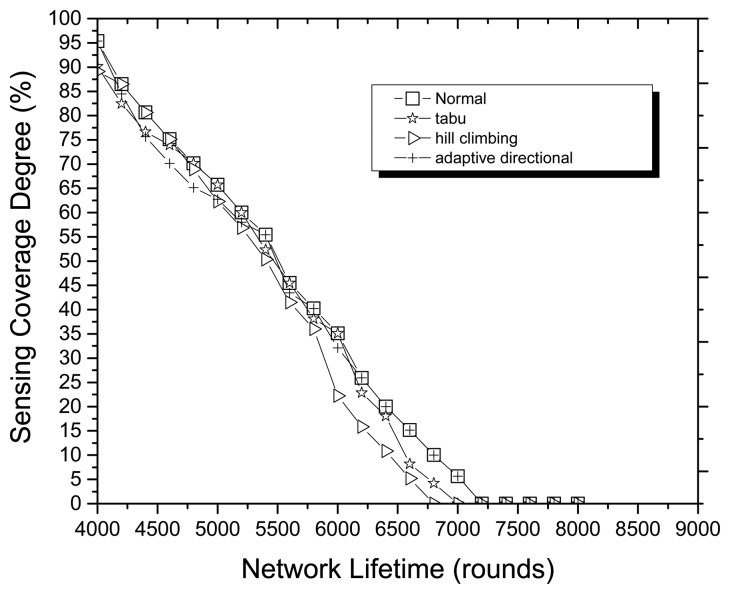
Performances of the dynamic local search strategy in the different cases.

Finally, we analyze the dynamic local search strategy of MOCOAMA through the simulation experiments with the same conditions as the previous experiments. In the normal case, there are tabu search algorithm, hill climbing search algorithm and adaptive directional local search strategy in the pool of local search algorithms, but when only one of those algorithms is in the pool, MOCOAMA would have a different performance. Figure 9 shows the performances of the dynamic local search strategy in the different pools, and the normal case of MOCOAMA has a better SCD than other cases. The better performance in the normal case may be due to the fact that MOCOAMA can dynamically use the advantages of the multiple local search algorithms.

## 7. Conclusions

This paper presents a multi-objective coverage optimization algorithm for WSNs, called MOCOAMA, which uses a memetic algorithm and gets the Pareto optimal solutions of the coverage problem. MOCOAMA maps the alternative solutions into the chromosomes represented as the matrices and uses a fitness function over sensing coverage degree, network coverage, energy consumption and Pareto ranking. We also introduce the dynamic local search strategy and a local algorithm scheduling mechanism to improving some or all chromosomes in one population. The simulation results show that MOCOAMA can solve the 1-coverage multi-objective problems for WSNs, get optimal network coverage, effectively extending the network lifetime, and have a significant improvement over some related algorithms even when the sensing error exists in the network.

In real applications, MOCOAMA depends on the sink which is assumed to have unlimited energy and finishes most of the problem-solving work according to the data sent from the sensor nodes, so how to reduce the dependence on the sink in designing memetic algorithm-based coverage algorithm shall be a challenging research task in the future.

## Figures and Tables

**Table 1.** Parameters used in simulations

**Parameter**	**Value**
Network size: n	≤ 400
Number of points of interest	64
Crossover probability: pcross	0.6
Mutation probability: pmutation	0.1
Maximum iterations	≤ 8000
Communication radius	20 m∼80 m
Packet length: *K*	300 bit
Number of clusters: *C*	≤ 40
Node initial energy: *E*_0_	10 J
Maximum ideal sensing radius: r*_i_*	15 m
Sensing error value: r*_e_*	≤1 m
λ	1
*E_elec_*	50 nJ/bit
*ε_fs_*	100 pJ/bit/m^2^
*E_DA_*	5 nJ/bit
*ε_amp_*	0.0013 pJ/bit/m^4^
*δ*_1_	1
*δ*_2_	1
